# Concurrent chemoradiation of metastases with capecitabine and oxaliplatin and 3D-CRT in patients with oligometastatic colorectal cancer: results of a phase I study

**DOI:** 10.1186/1748-717X-7-83

**Published:** 2012-06-09

**Authors:** Kathrin Dellas, Thomas Reese, Michael Richter, Dirk Arnold, Jürgen Dunst

**Affiliations:** 1 North European Radiooncological Center Kiel (NRoCK), Kiel, Germany; 2 Department of Radiooncology, University of Lübeck, Lübeck, Germany; 3 Department of Radiotherapy, Martin Luther University Halle-Wittenberg, Halle (Saale), Germany; 4 Coordination Center for Clinical Trials, Halle (Saale), Germany; 5 Hubertus Wald Tumor Center, University Comprehensive Cancer Center Hamburg-Eppendorf (UCCH), Hamburg, Germany

**Keywords:** Oligometastatic colorectal cancer, Chemoradiation, Capecitabine, Oxaliplatin, Phase I study

## Abstract

**Background:**

Local control appears to be an important treatment aim in patients with limited metastases (oligometastases) of colorectal cancer (CRC). Those patients show a favourable prognosis, if - in addition to the local effective treatment - an occurrence of new metastases may also be postponed by effective systemic therapy. The purpose of this dose escalation phase I study was to establish the efficacy of local radiotherapy (RT) of oligometastatic CRC with a concurrent standard chemotherapy regimen.

**Methods:**

Patients with first-, second- or third-line therapy of oligometastatic CRC (1–3 metastases or local recurrence plus max. 2 metastases) received capecitabine (825 mg/m^2^/d BID d 1–14; 22–35) and oxaliplatin (50 mg/m^2^ d 1, 8, 22, 29). 3D-conformal RT of all metastatic lesions was delivered in 2.0 Gy up to 36 Gy to 50 Gy (3 dose levels). Primary endpoint was the maximal tolerable dose (MTD) of RT defined as the level at which two or more of six patients experienced dose-limiting toxicity (DLT).

**Results:**

Between 09/2004 and 08/2007, 9 patients (7 male, 2 female, 50–74 years) were enrolled, 6 patients treated at dose level 1 (36 Gy), 3 patients at dose level 2 (44 Gy). 1 patient from the first cohort experienced DLT (oxaliplatin-related hypersensitivity reaction). No radiation-induced DLT occurred. 6/9 patients achieved objective response (partial remission). One year after initiation, all patients were alive, 6 patients survived (16 to 54 months) patients died of tumor progression (14 to 23 months). The phase II part of the trial had to be closed due to recruitment failure.

**Conclusions:**

Local 3D-CRT to metastatic lesions in addition to standard chemotherapy was feasible, DLT was not documented. 3/9 patients survived for a period of 3.5 to 4.4 years (time at the last evaluation). Radiotherapy of metastatic lesions should be incorporated into subsequent trials.

## Background

Colorectal cancer is one of the most common cancer diagnosis among both genders and with an estimated number of 207 400 (12.2%) of total deaths the second major cause of cancer death in Europe in 2006 [1]. At the time of diagnosis about 25% of patients present with metastases and more than one third of patients will develop metastatic disease after curative resection of the primary tumor in the further course of disease, mainly liver metastases.

Patients with a single or few liver or lung metastases should undergo curative intended resection of their metastases and have a chance of long-term cure in the range of 30 to 40% [2]. In irresectable metastases, palliative chemotherapy aims to prolong survival while preserving or improving the quality of life. However, definition of a potentially curative and a palliative approach has therefore been mainly determined by resectability in the past. Recently, a variety of non-surgical local ablative therapies have been developed, and the question was raised whether a subset of patients with a limited number of metastases (oligometastatic disease) might benefit from the addition of such local therapy to systemic chemotherapy.

Local efficacy of radiotherapy has been well documented in mCRC and palliative radiotherapy can be used effectively for controlling symptoms [3-8]. Though improved diagnosis and therapy planning also multiple lesions can be localized exactly and irradiated in small volume, respectively. A requirement for this is also a planning target definition adapted to the metastases. The combinations of oxaliplatin with capecitabine have shown both, efficacy in mCRC as systemic treatment and radiosensitizing potential [9-15], and are therefore suitable from a radiobiological point of view particularly for the combination with radiotherapy. The application can be used concurrently with radiotherapy, without increasing toxicity of the chemotherapy or radiotherapy considerly.

Therefore, the purpose of this dose escalation phase I study was to establish a regimen of local radiotherapy with concurrent standard chemotherapy in oligometastatic colorectal cancer.

## Patients and methods

The study was conducted according to the principles of the Declaration of Helsinki and to good clinical practice guidelines. The Ethics Committee, University of Halle, approved this study. Each patient gave written informed consent before being included.

### Eligibility criteria

The eligibility criteria included histopathologically confirmed oligometastatic colorectal cancer (1–3 metastases with largest diameter 5 cm or local recurrens of rectal cancer plus 1–2 metastases of ≤ 5 cm in technically or clinically irresectable disease, extention to the recruitment of patients in a neoadjuvant situation (before planned resection). Patients with first-, second- or third-line therapy of oligometastatic colorectal cancer were eligible for this study in adequate renal, hepatic and hematologic function. Additional inclusion criteria were age ≥ 18 years**,** Karnofsky performance status ≥ 70%**,** creatinin clearance > 30 mL/min calculated according to Cockroft and Gault, total bilirubin concentration less than 2.5 and transaminases less than 2.5 times the upper normal limit with hepatic metastases after image-guided exclusion of intra- or extrahepatic cholestasis and transaminases less than 5.0 times the upper normal limit, neutrophils > 2.5 x 10^9^/L, platelet count > 125 x 10^9^/L**,** estimated life expectancy of more than 3 months**,** written informed consent and tumor assessment analyzed according to “Response Evaluation Criteria in Solid Tumors” (RECIST).

Key exclusion criteria included previously administered radiotherapy not allowing the required dose, significant cardiac disease (heart failure NYHA III-IV, myocardial infarction within the last three months or symptomatic heart disease) and cerebrovascular disorders thought to adverse-affects treatment compliance. Patients with the following conditions were also ineligible: serious, uncontrolled infections, malabsorption syndrome, known sensitivity to fluoropyrimidines and dihydropyrimidine dehydrogenase deficiency, allergy to platin derivative, peripheral neuropathy, current treatment with sorivudin or bivudin and treatment history of other cancer or participation in another clinical trial within 4 weeks of the start of treatment. Pregnant or lactating patients and women with childbearing potential who lacked a reliable contraceptive method were also excluded.

### Study design and treatment

We undertook a prospective, single center phase I study. The primary endpoint was to determine the maximal tolerable dose (MTD) and dose-limiting toxicity (DLT) of chemoradiotherapy with concurrent 3D-radiotherapy to all (max. 3) metastatic lesions. Radiation dose levels of all metastatic leasions were the planned dose levels at 36 Gy, 44 Gy and 50 Gy in 2.0 Gy daily fractions (as described later, the dose level at 50 Gy was not reached). The secondary endpoints included the evaluation of antitumor activity of the combined-modality treatment in objective response according to the Response Evaluation Criteria In Solid Tumors (RECIST) [16] (rate of complete or partial remission), time to progression, overall survival (one year survival rate) and toxicity.

During radiotherapy, capecitabine was administered with a fixed dose (825 mg/m^2^/d orally BID on days 1–14 and 22–35), and oxaliplatin (50 mg/m^2^ as 2 h infusion on days 1, 8, 22 and 29). After completion of chemoradiation, systemic treatment could be continued in standard dosage (capecitabine 1000 mg/m^2^/d orally BID on days 1–14 and oxaliplatin 70 mg/m^2^ infusional on days 1 and 8). Staging was completed in the third week of the third cycle (week 9), and therapy was considered to continue at the physician’s discretion.

The following recommendations for dose reductions were applied: if one patient experienced grade 1 toxicity (according to National Cancer Institute Common Toxicity Criteria version 3.0) considered to be possibly related to radiation, treatment was continued, with appropriate prophylactic treatment. In case of grade 2 toxicity, radiation treatment was continued for one week at maximum. If patient experienced any grade 2 toxicity for more than one week or more severe intensity, radiotherapy was halted for 3–7 days. When toxicity resolved to grade 1, treatment was resumed, if possible. Interruption or conduction of radiation may be related to one or several planning target volumes. In case of recurrence of toxicity while continuing radiation, which would require further discontinuation of radiation therapy (as defined above) radiation of this planning target volume was permanently discontinued. Radiotherapy was considered to be in accordance with the protocol if at least 80% of the planed dosage for the planning target volume was applied and therapy was no longer discontinued than for 7 days.

### 3D-CRT technique

A total irradiation dose of 36 Gy or 44 Gy was delivered in 2.0 Gy daily fractions, Monday through Friday. In this protocol, high-energy photons (6 to 15 MeV) were used and three-dimensional planning with measurements of macroscopic tumor, planning target volume and organs at risk were mandatory. All lesions had to be defined by computed tomography clearly. The clinical target volume (CTV) was the GTV of each lesion. Planning target volume contained all detectable metastases was derived by adding a margin of 10 mm to account the CTV for the setup uncertainties. It was required that the 90%-isodose line covered the planning target volume. Two metastatic lesions could be integrated into one planning target volume if they were located close to each other.

### Evaluation of safety and efficacy

Adverse events were graded to National Cancer Institute of Canada (NCIC) Common Toxicity Criteria (CTC) (revised in May 1991). Dose-limiting toxicity (DLT) was defined as the occurrence of any grade three and four toxicity, except alopecia, hematologic toxicity and transient elevation of transaminases. Within the three dose levels, dose escalation of radiation was performed with the same dose of chemotherapy. According to potentially different toxicity at different sites of metastatic lesions, modified rules for escalation were used: Three patients had to be enrolled per predefined dose level. If there was no DLT in the first three patients, the cohort was extended by another three patients at the same dose level, to obtain sufficient safety profile associated with different toxicity depending on multiple metastatic lesions.

The safety analysis included all patients who received at least one dose (one day) of radiation and chemotherapy. All adverse events were monitored continuously during treatment and observed until decreasing or stabilization of symptoms. Hematology and clinical chemistry was performed weekly during treatment and thereafter during chemotherapy weekly, respectively after every third cycle, after the end of chemotherapy and during follow up every three months. Tumor assessment was initiated on the basis of RECIST criteria 1.0 at baseline, within 14 days before the start of treatment, and after the end of the combined treatment.

### Statistical aspects

A modified escalation design with three to six patients was chosen on empiric grounds, according to current standards in phase I cancer trials. According to the exploratory nature of this pilot trial, only descriptive statistical methods are used, giving rates, means with SD, and quartiles and ranges.

## Results

A total of nine patients were enrolled (seven male and two female patients between 50 and 74 years of age) into the study at Halle University between September 2004 and August 2007 (Table 1). Before including into the study, six patients had been previously treated with radiotherapy at other sites, and all of the nine patients received previously chemotherapy and surgery. In three patients, systemic pretreatment was administered as capecitabine and oxaliplatin, one patient received capecitabine, oxaliplatin, 5-FU and leucovorin, one patient received 5-FU and leucovorin, one patient received 5-FU and oxaliplatin, one patient was treated with 5-FU, leucovorin, oxaliplatin and irinotecan, one patient was treated with 5-FU, leucovorin, capecitabine, oxaliplatin, irinotecan and cetuximab and one patient received 5-FU, leucovorin, irinotecan and bevacizumab. Issued by the criteria for inclusion the metastatic lesions were not restricted to the liver. The number of metastatic lesions was a single lesion in four patients, two lesions in two patients and three lesions in three patients (a total of 17 lesions in 9 patients). In seven patients the lesion was located in the liver and two patients had a non-liver-lesion (vulva and local recurrence presacral).

**Table 1:** Characteristics of patients

		Dose level 1 n = 6	Dose level 2 n = 3	Total n = 9
Sex				
Male		4	3	7
Female		2	-	2
Age, years				
MW		63.2	65.3	63.9
SD		10.1	5.0	8.4
Karnofsky performance status (%)				
MW		93.3	96.7	94.4
SD		8.2	5.8	7.3
Pathologic staging (at the time of accrural)				
T	T0	4	3	7
	T4	2	-	2
N	N0	5	3	8
	N1	1	-	1
M	M0	1	-	1
	M1	5	3	8

MW: Mean, SD: Standard deviation.

### Dose escalation and DLT

Initially, three patients were treated at the lowest dose level of radiation (36 Gy), without dose-limiting toxicity. According to the study protocol, three additional patients were included at this dose level. Therefore, six patients were treated at the lowest dose level one and three patients at dose level two (44 Gy). The fourth patient from the first cohort experienced a non-radiotherapy related DLT (oxaliplatin-related hypersensitivity reaction). There was no DLT in the three patients treated in the second cohort until the premature termination. No radiation-induced DLT occurred. The dosage finding remained incomplete and MTD could not be determined. The trial had to be closed due to recruitment failure. There were no safety-related concerns regarding the premature trial termination.

### Hematologic and non-hematologic toxicity

Adverse events according to MedDRA Code CTC severity grade by body/organ system are presented for the whole patients group (Table 2). The only grade 4 toxicity was oxaliplatin-related hypersensitivity reaction (one patient in cohort one), consecutively classified as DLT. Four patients experienced grade 3 toxicity, three patients from the first cohort and one patient from the second dose level. In the three patients at dose level one, grade 3 toxicities occurred (infection, dyspnea, pain and leucocytopenia). Grade 3 toxicity with increasead liver enzymes was documented in one patient at dose level two. No instance of radiation-induced liver disease (RILD) has been observed.

**Table 2:** Incidence and maximum severity of toxicities (MedDRA classification)

	Dose level 1			Dose level 2		∑		
CTC grade	1/2	3	4	1/2	3	1/2	3	4
MedDRA Code (n = 9 events)	No. of Patients							
Infections and infestations								
Infection	0	2	0	0	0	0	2	0
Immune system disorders								
Anaphylactic reaction	0	0	1	0	0	0	0	1
Nervous system disorders								
Peripheral sensory neuropathy	2	0	0	1	0	3	0	0
Eye disorders								
Conjunctival haemorrhage	1	0	0	0	0	1	0	0
Vision blurred	1	0	0	0	0	1	0	0
Disorder sight	1	0	0	0	0	1	0	0
Abnormal sensation in eye	1	0	0	0	0	1	0	0
Vascular disorders								
Flushing	2	0	0	0	0	2	0	0
Hypotension	1	0	0	1	0	2	0	0
Thrombophlebitis	1	0	0	0	0	1	0	0
Respiratory, thoracic and mediastinal disorders								
Dyspnoea	0	1	0	0	0	0	1	0
Gastrointestinal disorders								
Abdominal pain	0	0	0	1	0	1	0	0
Abdominal distension	1	0	0	0	0	1	0	0
Vomiting	0	0	0	1	0	1	0	0
Obstipation	2	0	0	0	0	2	0	0
Mucous stools	1	0	0	0	0	1	0	0
Nausea	3	0	0	2	0	5	0	0
Abdominal discomfort	1	0	0	0	0	1	0	0
Epigastric discomfort	0	0	0	1	0	1	0	0
Skin and subcutaneous tissue disorders								
Dermatitis	1	0	0	2	0	3	0	0
Musculoskeletal and connective tissue disorders								
Bone pain	1	0	0	0	0	1	0	0
Myalgia	1	0	0	0	0	1	0	0
Pain in extremity	1	0	0	0	0	1	0	0
Renal and urinary disorders								
Bladder discomfort	2	0	0	0	0	2	0	0
General disorders and administration site conditions								
Injection site erythema	0	0	0	1	0	1	0	0
Pyrexia	1	0	0	1	0	2	0	0
Injection site paraesthesia	0	0	0	1	0	1	0	0
Mucosal inflammation	1	0	0	0	0	1	0	0
Pain	3	1	0	1	0	4	1	0
Chills	1	0	0	1	0	2	0	0
Investigations								
Blood amylase increased	1	0	0	0	0	1	0	0
Blood bilirubin increased	2	0	0	3	0	5	0	0
Blood pressure increased	0	0	0	2	0	2	0	0
Blood urea increased	1	0	0	0	0	1	0	0
Blood urea decreased	0	0	0	2	0	2	0	0
C-reactive protein increased	4	0	0	1	0	5	0	0
Blood fibrinogen increased	1	0	0	0	0	1	0	0
Gamma-glutamyltransferase increased	4	0	0	1	1	5	1	0
Blood glucose increased	0	0	0	1	0	1	0	0
Haemoglobin decreased	4	0	0	2	0	6	0	0
Blood uric acid increased	1	0	0	0	0	1	0	0
Blood calcium decreased	3	0	0	2	0	5	0	0
Blood creatinine increased	3	0	0	1	0	4	0	0
Blood lactate dehydrogenase increased	3	0	0	1	0	4	0	0
WBC count increased	1	0	0	0	0	1	0	0
WBC count decreased	1	1	0	2	0	3	1	0
Platelets decreased	3	0	0	2	0	5	0	0
Transaminases increased	2	0	0	3	0	5	0	0

Further hematologic and non-hematologic toxicity was usually mild (grade 1 and 2). Five patients showed any increased levels of blood bilirubin, transaminases and C-reactive protein, six patients an elevation of gamma-glutamyltransferase and four patients of lactate dehydrogenase. Low hemoglobin as well as low platelet counts were seen in six and five patients, respectively. Gastrointestinal toxicities occurred in five patients consisting primarily of nausea and in two patients with abdominal and epigastric discomfort. Furthermore, dermatitis was recorded in three patients.

### Antitumor activity

Secondary endpoint was the objective tumor response, defined as the rate of complete or partial remission. A total of seventeen lesions in nine patients were irradiated. Six of nine patients achieved tumor response (partial remission), for two patients adequate data were not assessable. Disease progression after one year occurred in three patients (two patients from the first cohort, one patient from the second cohort). The first patient from the first cohort developed new liver metastases and distant metastases (lung lesions). For the second patient from the first cohort and the patient from the second cohort with progressive disease adequate data were not available. One year after initiation, all patients were still alive. Within the following year, three patients died from tumor progression (14 to 23 months after initiation). At the time of last evaluation four patients had survived (16 to 54 months after initiation, Table 3).

**Table 3:** Parameters for survival

	Dosis 1		Dosis 2		∑	
	Survived	Lost to Follow up	Survived	Lost to Follow up	Survived	Lost to Follow up
1 year	6	0	3	0	9	0
3 years	3	0	0	3	3	3

One year after initiation all patients were alive, three patients had died of tumor progression within the following three years.

## Discussions

We conducted this phase I trial as a multimodal regimen for patients with oligometastatic colorectal cancer, consisting of 3D-radiotherapy of all metastatic lesions with a concurrently administered standard systemic therapy with capecitabine and oxaliplatin (XELOX regimen) [17,18]. The primary objective of this study was to determine the maximal tolerable dose (MTD) of local radiotherapy combined with standard chemotherapy. The study used conventionally fractionated radiotherapy under the assumption that this treatment is widely available, safe and effective as known from preoperative radiotherapy in locally advanced rectal cancer.

Most commonly, local disease control can be assessed by pathohistological response, which is likely correlated with relapse free suvival after 5-FU based chemoradiotherapy. Pathological complete response (pCR) rates following fluoropyrimidine based chemoradiation have been reported in 15-20% of patients. Many phase II trials indicate, that the rate of pathohistologic response (pCR) of the local tumor may be improved by adding highly effective substances like oxaliplatin, combined with 5-FU or capecitabine - an emerging strategy in the multimodality management of locally advanced rectal cancer [19]. In preoperative chemoradiation for localized rectal cancer, phase II and III trials have shown that the combination of radiation with capecitabine and oxaliplatin shows moderately high rates of histopathological eradication of the tumor. Data of phase II trials suggests a pCR rate up to the above mentioned 20%, whereas rates in phase III trials only showed “borderline” significant improvement [12-15]. The arguments for a regimen of chemoradiation, based on capecitabine, oxaliplatin and radiation, are the efficacy of the chemoradiation, radiosensitizing potential and the approved feasibility of the mentioned combined regimen in terms of systemic activity. However, the final prove of effectiveness of adding oxaliplatin to the preoperative therapy is still under investigation, but reported preliminary results from phase III trials, the French ACCORD study [12], the Italian STAR-01 study [15] as well as the US NSABP R-04 trial [14] did not show a significant increased rate of pCR´s, and in the German phase III trial, only a small increment was documented [13]. It has to be shown, whether these results impact on lead to improved local failure or distant metastases.

We aimed to evaluate the efficacy and feasibility of adding capecitabine and oxaliplatin, administered at fixed doses according to a schedule previously developed in rectal cancer [17,18], in addition to dose escalation of radiation of multiple metastatic lesions in patients with mCRC.

There are several limitations of the study with respect to the efficacy caused by the inhomogenous characteristics of patients, the patients (pre)treatment with the first-, second- or third-line therapy and incomplete information of local lesions and distant metastases of patients with progressive disease.

Notwithstanding the trial had to be closed due to recruitment failure, and the small sample size limits conclusions. However, the data are promising with regard to the results of the present trial and should stimulate more intense investigations to establish the best treatment regimens to obtain better approaches in the therapy of oligometastatic colorectal cancer. Moreover, more advanced radiotherapy techniques (e.g. IGRT, stereotactic radiotherapy and radiosurgery) are now widely available and 3D-CRT is not considered as contemporary standard for radiotherapy to metastatic lesions in a potentially curative setting. Nevertheless, our results might be helpful because they demonstrate the feasibility of a concurrent chemoradiotherapy approach in this subset of patients and there are so far few data on the question of sequencing local and systemic therapy in metastatic patients.

## Conclusions

The results of this phase I study support the use of local radiotherapy to metastatic lesions in addition to concurrent standard chemotherapy as an effective and feasible therapeutic option in patients with oligometastatic colorectal cancer.

## Competing interests

The authors declare no conflicts of interest.

## Authors’ contribution

KD, MR, DA and JD designed and supervised the study. KD, TR, DA and JD responsible for therapy and data collection. MR collected data and did statistical analyses. KD drafted the manuscript and figures, and all authors edited and/or approved the final version.
